# Construction of circRNA‐based ceRNA network and its prognosis‐associated subnet of clear cell renal cell carcinoma

**DOI:** 10.1002/cam4.4311

**Published:** 2021-09-27

**Authors:** Yuwei Zhang, Yuchen Zhang, Yangkun Feng, Nan Zhang, Saisai Chen, Chaoqun Gu, Lei Hu, Jiayi Sheng, Bin Xu, Ninghan Feng

**Affiliations:** ^1^ Department of Urology Affiliated Wuxi No. 2 Hospital of Nanjing Medical University Wuxi China; ^2^ Department of Oncology First Affiliated Hospital of Nanjing Medical University Nanjing China; ^3^ Medical College of Nantong University Nantong China; ^4^ Department of Urology Affiliated Zhongda Hospital of Southeast University Nanjing China; ^5^ Southeast University Nanjing China

**Keywords:** CCDC8, ceRNA network, circRNA, clear cell renal cell carcinoma (ccRCC), has_circ_0001167, prognosis

## Abstract

Circular RNAs (circRNAs) are novel biomarkers of various cancers. CircRNAs can sponge miRNAs and regulate target mRNAs, which was called competing endogenous RNAs (ceRNA). This study was designed to identify circRNAs related to patients with clear cell renal cell carcinoma (ccRCC) and the first to select three independent Gene Expression Omnibus microarrays covering circRNAs, miRNAs, and mRNAs for multiple analyses. The data of clinical cases applied in our study were obtained from The Cancer Genome Atlas. We successfully conducted a circRNA/miRNA/mRNA ceRNA network related to ccRCC patients via R software and Cytoscape including 8 circRNAs, 6 miRNAs, and 49 mRNAs. The prognosis‐associated subnet covered 8 circRNAs, 6 miRNAs, and 22 mRNAs. Quantitative real‐time PCR was applied to measure our prediction in three renal cell lines and 23 pairs of tissues. Small interfering RNA targeting the back‐splice region of hsa_circ_0001167 was further implied to confirm the regulation. Ultimately, hsa_circ_0001167/hsa‐miR‐595/CCDC8 regulatory axis was identified in this study, which may serve as prognostic indicators. Lower levels of hsa_circ_0001167 and CCDC8 were potentially correlated with worse patient survival.

## Graphical abstract

Based on the analyzation of three Gene Expression Omnibus microarrays and The Cancer Genome Atlas cases, we were the first to construct a ccRCC centred ceRNA network and its prognosis‐associated subnet. We further validated a hsa_circ_0001167/ has‐miR‐595/CCDC8 axis in renal cell lines, which may serve as prognostic indictors.

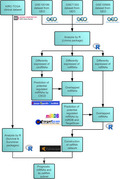

## 1 INTRODUCTION

Renal cell carcinoma (RCC) is one of the most prevalent malignant diseases worldwide, accounting for 2%–3% of new cancers each year.^1^ The histological types of RCC consist of three categories, including clear cell renal cell carcinoma (ccRCC), chromophobe RCC, and papillary RCC.^2^ Among them, ccRCC is the most common type of kidney cancer pathology, accounting for 75% of all pathological types of RCC.^3^ Although surgery, targeted therapies, and various immunotherapy agents have achieved certain effects in patients with RCC,^4^ the 5‐year survival rate for ccRCC patients remains 12%,^5^ highlighting the need to discover better prognostic indicators.

In recent years, the importance of non‐coding RNAs (ncRNAs) in the occurrence and development of ccRCC has received increasing attention.^6^ Circular RNA (circRNA) is a new type of ncRNA derived from the precursor mRNA. CircRNAs uniquely exhibit a covalently closed‐loop structure, which confers higher stability and potential as biomarkers.^7^ A large number of microRNAs (miRNAs) have been found to play an important role in cancer progression, such as hsa‐miR‐206 and hsa‐miR‐101, which were identified as important in the biological process (BP) of ccRCC development.^8^, ^9^, ^10^ It has been demonstrated that some circRNAs possess miRNA binding sites and function as miRNA sponges, which can suppress the activity of miRNA and regulate the expression of target genes.^11^, ^12^ This is known as the competitive endogenous RNA (ceRNA) regulatory system. Many studies have proved that circRNAs regulate mRNAs through the ceRNA network to take part in the progression of ccRCC. For instance, Liu et al.^13^ demonstrated that the circ‐PTCH1/miR‐485‐5p/MMP14 axis promoted RCC metastasis. Xue et al.^6^ showed that circAKT3 competitively combined miR‐296‐3p to inhibit ccRCC metastasis.

In this study, a ceRNA network (circRNA/miRNA/mRNA) and its prognosis‐associated subnet were constructed to investigate the potential pathogenesis of ccRCC and provide a predictive value for the prognosis of patients with ccRCC.

## 2 MATERIALS AND METHODS

### 2.1 Data obtained

The profiles of circRNA, miRNA, and mRNA for human samples derived from patients with ccRCC were obtained from the GEO database (https://www.ncbi.nlm.nih.gov/geo/). We selected the GSE100186 circRNA microarray, GSE71302 miRNA microarray, and GSE100666 mRNA microarray based on preset criteria: (1) The expression profiles of circRNA, miRNA, and mRNA in human samples were derived from ccRCC patients and matched normal tissues; (2) Candidate microarrays enrolled at least three pairs of samples; (3) microarrays for more than 5 years will be discarded. The GSE100186 circRNA microArray, GSE71302 miRNA microArray, and GSE100666 mRNA microArray were based on the platform GPL21825 074301 Arraystar Human CircRNA microarray V2, GPL10850 Agilent‐021827 Human miRNA Microarray (V3) (miRBase release 12.0 miRNA ID version), and GPL16951 Phalanx Human OneArray Ver. 6 Release 1 respectively. The KIRC‐TCGA clinical dataset was extracted from TCGA gdc (https://portal.gdc.cancer.gov/) on 20 September 2020, which included 537 cases. The results of immunohistochemical expression of CCDC8 in ccRCC tissues and normal tissues were acquired from HPA database (https://www.proteinatlas.org/).

### 2.2 Acquisition of differentially expressed circRNAs, miRNAs, and mRNAs

Normalization and log2 transformation were performed on all raw data. "Limma" package of Bioconductor in R software (version 4.0.2) was applied to correct the microarray data to acquire DECs, DEmiRs, and DEmRs with the unified criteria of |log2 fold change| >1 and adjusted *p* value (*q* value) <0.05.

### 2.3 Prediction of target miRNAs and mRNAs

With the help of CSCD (https://gb.whu.edu.cn/CSCD/), we visualized the circRNAs and predicted the MRE, ORF, and RBP. Databases of TargetScan (http://www.targetscan.org/mamm_31/) and miRDB (http://mirdb.org/) were crossed to forecast miRNA target mRNAs.

### 2.4 Construction of a ceRNA network

First, DECs were found by analyzing circRNA microarray downloaded from GEO. The 1059 DECs were sorted from high to low according to their fold changes, and the top 100 DECs were selected for subsequent analyses. Then we overlapped the MREs predicted by CSCD and DEmiRs from miRNA microarray to ensure candidate miRNAs. MiRDB and TargetScan were two databases we applied to predict miRNA target mRNAs, which were further overlapped with DEmRs analyzed from mRNA microarray. We examined the differential expression of the 58 circRNAs, 11 miRNAs, and 204 mRNAs and further screened them according to the regulatory relationship of ceRNA. The circRNA/miRNA/mRNA ceRNA network was finally constructed.

### 2.5 Construction of a prognosis‐associated subnet

The KIRC‐TCGA clinical dataset were acquired from TCGA gdc (https://portal.gdc.cancer.gov/) on 20 September 2020, which included 537 cases. We extracted the TCGA data corresponding to 49 genes of ceRNA network and analyzed them one by one. Patients were divided into two groups such as high‐expression group and low‐expression group according to the expression of each gene. Data about the survival time of two groups were further analyzed. “Survival” and “Survminer” were two packages of R software we utilized and the results were displayed by K‐M survival curves. The gene was identified as a prognosis‐associated gene when the *p* value was smaller than 0.05. Finally, we identified 22 significant genes among 49 genes of ceRNA network, according to which, a prognosis‐associated subnet was constructed.

### 2.6 GO and KEGG analyses of mRNAs

Gene Ontology analysis and KEGG analysis were applied to analyze mRNAs in the network with a *p* value <0.05. GO analysis was utilized for functional analysis, which was consisted of BP, CC, and MF. KEGG pathway analysis implied signaling pathway information for the genes. All analyses were carried out by the “Bioconductor” package of R software.

### 2.7 Construction of PPI network

PPI networks were built based on information of the circRNA/miRNA/mRNA network and prognosis‐associated subnet with the help of the Search Tool for the Retrieval of the Interacting Genes (STRING, https://string‐db.org/).

### 2.8 Cell culture

The human renal cancer cell lines 786‐O, Caki‐1, and normal renal cell line HEK 293T cells were purchased from the Cell Bank of the Chinese Academy of Sciences. 786‐O, Caki‐1, and HEK 293T cell lines were cultured in RPMI 1640 medium (Gibco) mixed with 10% fetal bovine serum (BI); and 1% penicillin/streptomycin (Gibco) in a humidified atmosphere of 5% CO_2_ at 37℃. All experiments were carried out with mycoplasma‐free cells.

### 2.9 Oligonucleotide transfection

786‐O and Caki‐1 cells were seeded in six‐well plates and were transfected with siRNA when cultured to 60%–70%. SiRNA was transfected using Lipofectamine 2000 (Thermo Fisher Scientific) according to the manufacturer's protocol. The target sequences of siRNA are shown in Table S1.

### 2.10 RNA extraction and quantitative real‐time PCR

Total cellular RNA was extracted with Trizol Regent (Invitrogen). Reverse transcription was performed with HiScript III RT SuperMix (Vazyme) for circRNA and mRNA. As for miRNA, cDNA was synthesized using a HiScript II Q Select RT SuperMix (Vazyme). qRT‐PCR was utilized to assess the expression of circRNAs, miRNAs, and mRNAs, which was carried out in triplicate by HieffTM qPCR SYBR® Green Master Mix (Yeasen) and LightCycler® 96 SW 1.1 system (Roche). β‐Actin and U6 were used as endogenous controls for circRNA, mRNA and miRNA, respectively. The primers of circRNA and miRNA were designed by CircPrimer^14^ and MiRPrimer (Vazyme, http://www.vazyme.com/companyfile/251.html), respectively. As for mRNA, the primers were found in PrimerBank.^15^, ^16^, ^17^, ^18^ All primers were blasted on NCBI (https://www.ncbi.nlm.nih.gov/tools/primer‐blast/index.cgi?LINK_LOC=BlastHome) and the primers applied are displayed in Table S1. The comparative cycle threshold value (2‐ΔΔCT) was used to calculate results.

### 2.11 Statistical analysis

All analyses were performed with the help of R software (version 4.0.2). The independent *t* test was used to compare the continuous variables with normal distribution, and the Mann‐Whitney *U* test was used to compare the continuous variables with skewed distribution. All statistical tests were two‐sided.

## 3 RESULTS

### 3.1 Differentially expressed circRNAs (DECs), miRNAs (DEmiRs), and mRNAs (DEmRs) in ccRCC

**FIGURE 1:**
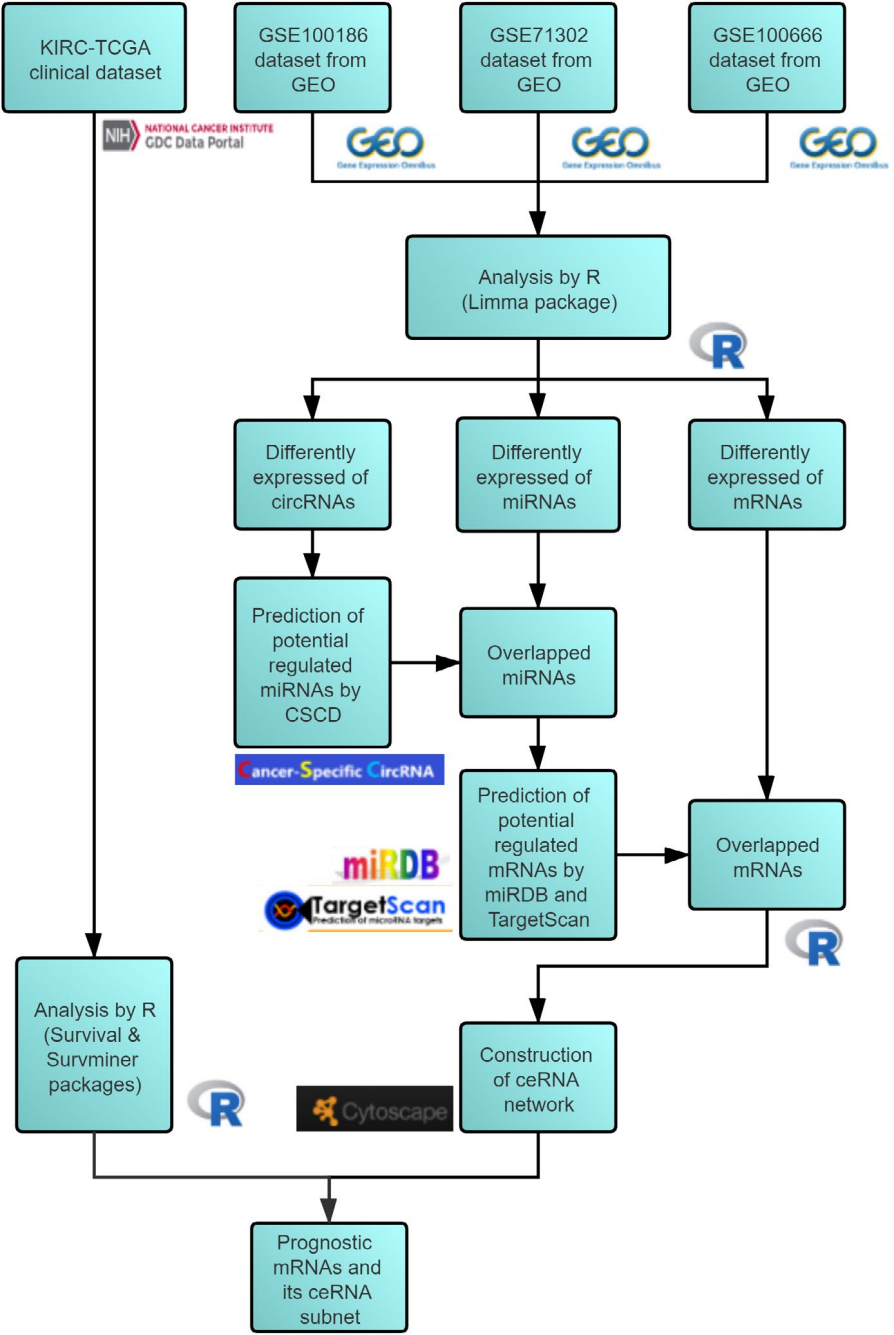
Flowchart of the study design

The layout of our study is displayed in Figure 1. Three microarray Gene Expression Omnibus (GEO) dataset were included in this study, as detailed in Table 1.

**TABLE 1:** Information about the three microarrays acquired from the Gene Expression Omnibus (GEO)

GEO accession	Contributor(s)	Year	Region	Tissue information	No. of genes
GSE100186	Lv Q, Wang P	2017	China	Four CCRCC tissues and matched non‐tumor tissues	10,140 circRNAs
GSE71302	Wang X, Zhang X	2015	China	Five CCRCC tissues and normal kidney tissues (NCTs)	851 miRNAs
GSE100666	Peng Z, He J, Zheng J	2017	China	Three ccRCC tissues and corresponding adjacent normal tissues	20,077 mRNAs

**FIGURE 2:**
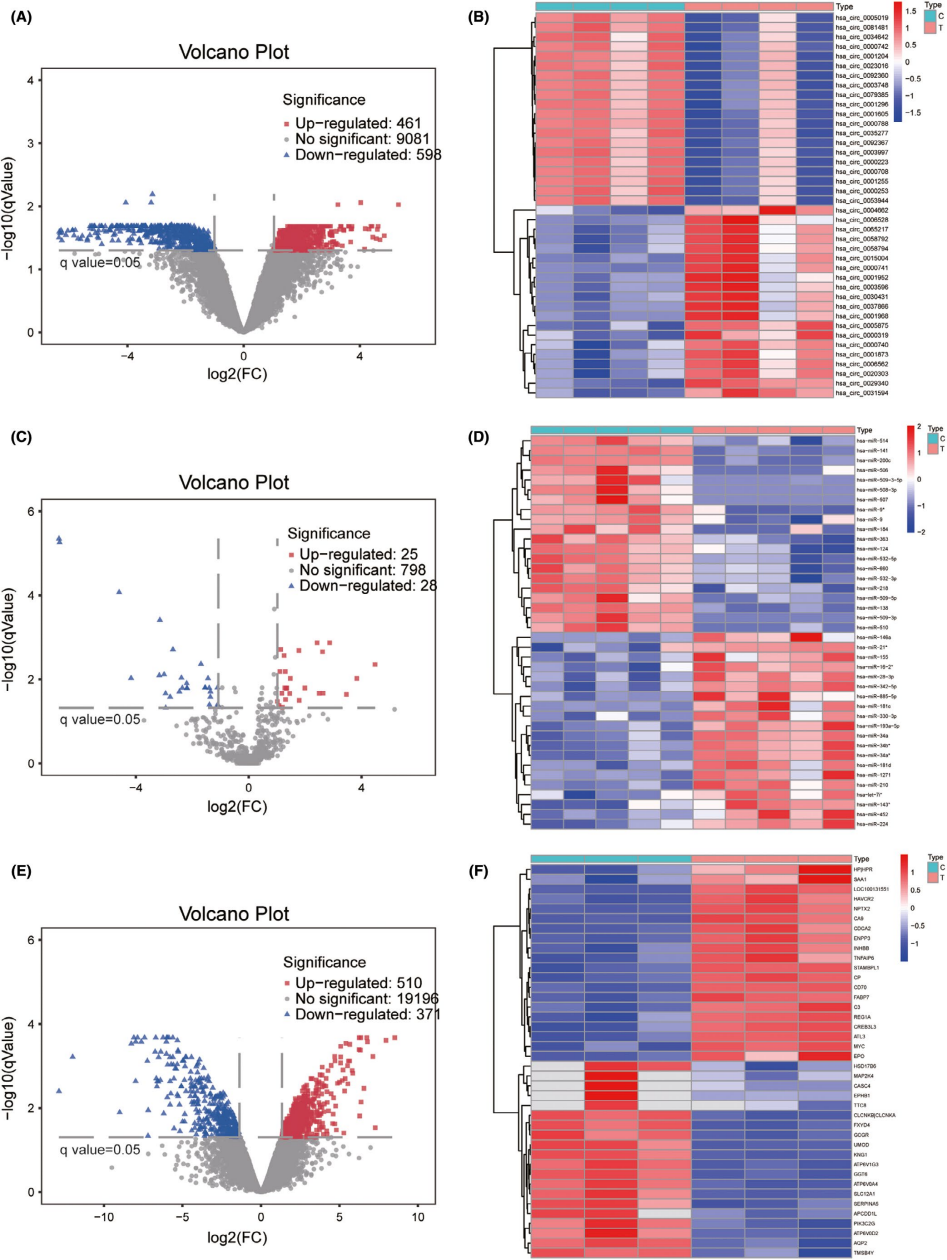
Identification of differentially expressed circRNAs (DECs), miRNAs (DEmiRs), and mRNAs (DEmRs). (A) Volcano plot for DECs. (B) Heatmap for top 20 up‐regulated and top 20 down‐regulated DECs. (C) Volcano plot for DEmiRs. (D) Heatmap for top 20 up‐regulated and top 20 down‐regulated DEmiRs. (E) Volcano plot for DEmRs. (F) Heatmap for top 20 up‐regulated and top 20 down‐regulated DEmRs. C: Normal tissues; T: Tumor tissues

The GSE100186 circRNA microarray contained four ccRCC tissues and four paired non‐tumorous tissues. The GSE71302 miRNA microarray included five ccRCC tissues and five matched normal kidney tissues. The GSE100666 mRNA microarray covered three ccRCC tissues at different stages and three normal tissues. In total, 1059 DECs, 53 DEmiRs, and 881 DEmRs were detected from GSE100186, GSE71302, and GSE100666 respectively. Volcano plots and heatmaps are shown to visualize the DECs (Figure 2A,B), DEmiRs (Figure 2C,D), and DEmRs (Figure 2E,F). Only the first 20 up‐regulated and 20 down‐regulated DECS, DEmiRs, and DEmRs are shown in the heatmaps.

### 3.2 Construction of a ceRNA (circRNA/miRNA/mRNA) network

The 1059 DECs were sorted from high to low according to their fold changes, and the top 100 DECs were selected for subsequent analysis. To visualize circRNAs and find their target miRNAs, 100 DECs were searched on Cancer‐Specific CircRNA database (CSCD), through which 58 circRNAs were identified, and a total of 1714 potential target miRNAs were recorded. After the intersection with the 53 DEmiRs from GEO, 11 miRNAs were finally identified (Figure S1A).

Targetscan (http://www.targetscan.org/mamm_31/) and miRDB (http://mirdb.org/) were crossed to predict the target mRNAs of above 11 miRNAs, through which 4310 potential target mRNAs were predicted. After crossing with 881 DEmRS, 204 mRNAs were eventually enrolled (Figure S1B).

**FIGURE 3:**
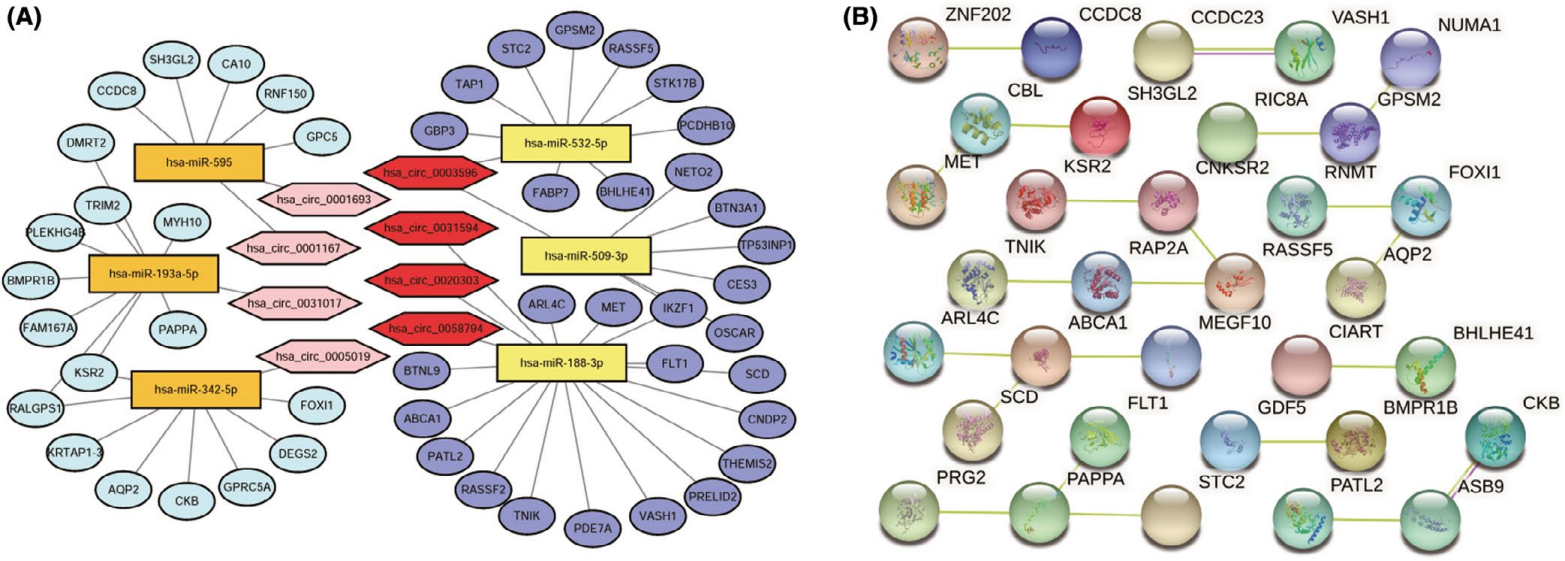
Construction of a circRNA/miRNA/mRNA ceRNA network and a PPI network. (A)The network consists of 8 circRNAs, 6 miRNAs, and 49 mRNAs. CircRNAs, miRNAs, and mRNAs are respectively represented by hexagons, rectangles, and ellipses. Relatively darker color indicates higher expression in the tumor tissues relative to normal tissues. (B) PPI network of genes in the ceRNA network. The lines in different colors represent different relationships (Pink line: experimentally determined; Yellow line: textmining)

We examined the differential expression of the 58 circRNAs, 11 miRNAs, and 204 mRNAs mentioned above in their GEO microarrays and further screened them according to the regulatory system of ceRNA.^11^, ^12^ With the help of Cytoscape (version 3.8.0), we successfully obtained the circRNA‐based ceRNA regulatory network (Figure 3A), which covered 8 circRNAs, 6 miRNAs, and 49 mRNAs. We then visualized the 8 circRNAs in Figure S2 by CSCD, including the miRNA response elements (MRE), open reading frames (ORF), and potential RNA binding proteins (RBP). Compared with normal tissues, 4 circRNAs, 3 miRNAs, and 29 mRNAs of the network were up‐regulated in ccRCC tissues, which were shown in darker colors. To observe the circRNAs, miRNAs, and mRNAs more visually, heatmaps (Figure S3A,C,E) and boxplots (Figure S3B,D,F) were constructed. The details (circBASE ID, circRNA position, type, the best transcript, strand, host gene, and length) of eight circRNAs are shown in Table 2.

**TABLE 2:** Information of eight circRNAs in the ceRNA network

circBASE ID	circRNA type	Position	Strand	Best transcript	Host gene	Length
hsa_circ_0001167	Intron	chr20: 47316514–47316617	−	NM_020820	PREX1	103
hsa_circ_0001693	Exon	chr7: 30590251–30601744	−	TCONS_00012989	TCONS_00012989	11,493
hsa_circ_0005019	Exon	chr15: 101775286–101775782	−	NM_014918	CHSY1	496
hsa_circ_0058794	Exon	chr2: 236626200–236659132	+	NM_001037131	AGAP1	451
hsa_circ_0020303	Exon	chr10: 125771848–125806240	−	NM_015892	CHST15	2007
hsa_circ_0031594	Exon	chr14: 34398281–34400421	−	NM_022073	EGLN3	257
hsa_circ_0003596	Exon	chr9: 137716445–137717750	+	NM_000093	COL5A1	369
hsa_circ_0031017	Exon	chr13: 114149816–114175048	+	NM_017905	TMCO3	1423

Note

CeRNA network: Some circRNAs possess miRNA binding sites and function as miRNA sponges, which can suppress the activity of miRNA and regulate the expression of target genes.

### 3.3 Functional annotation of the mRNAs

In order to analyze the function of the 49 mRNAs, we performed Gene Ontology (GO) and Kyoto Encyclopedia of Genes and Genomes (KEGG) analyses. GO analysis was utilized for functional analysis of the BP, cellular component (CC), and molecular function (MF).

**FIGURE 4:**
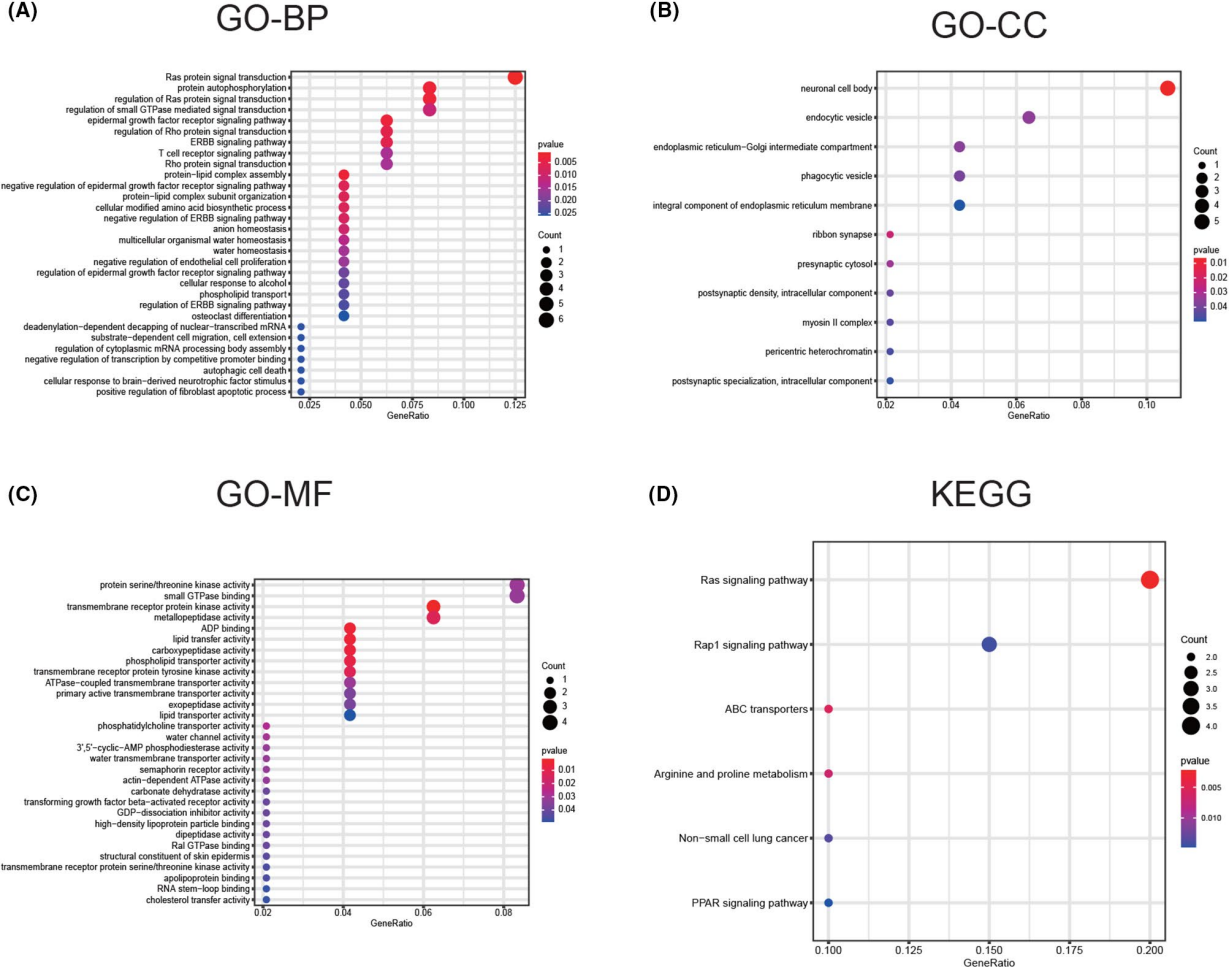
Functional annotation of the 49 mRNAs in the ceRNA network. (A) BP analysis. (B) CC analysis. (C) MF analysis. (D) KEGG analysis. Biological process (BP), cellular component (CC), molecular function (MF), and Kyoto Encyclopedia of Genes and Genomes (KEGG)

The 49 genes were related to the regulation of epidermal growth factor receptor (EGFR) signaling pathway, endothelial cell proliferation, cell migration, and autophagic cell death in BP (Figure 4A). With regard to CC (Figure 4B) and MF (Figure 4C), the 49 genes were involved in cellular energy metabolism, such as ADP binding, ATPase‐coupled transmembrane transporter activity, and actin‐dependent ATPase activity. The EGFR pathway, cell proliferation, migration, apoptosis and celluar energy metabolism were very important in the development of ccRCC.^19^, ^20^, ^21^, ^22^ As for KEGG analysis, non‐small cell lung cancer pathway was also identified, which further proved the close correlation of the 49 genes and cancers.

### 3.4 Construction of prognosis‐associated ceRNA subnet

Kidney renal clear cell carcinoma (KIRC) ‐TCGA clinical dataset was assessed from the TCGA gdc (https://portal.gdc.cancer.gov/) on 20 September 2020, which included 537 cases. Data about the expression of 49 mRNAs and the survival time of ccRCC patients were further analyzed. The results were displayed by Kaplan‐Meier (K‐M) survival curves. Finally, 22 mRNAs were determined as prognostically associated with ccRCC patients. All 22 K‐M survival curves are displayed in Figure S4.

**FIGURE 5:**
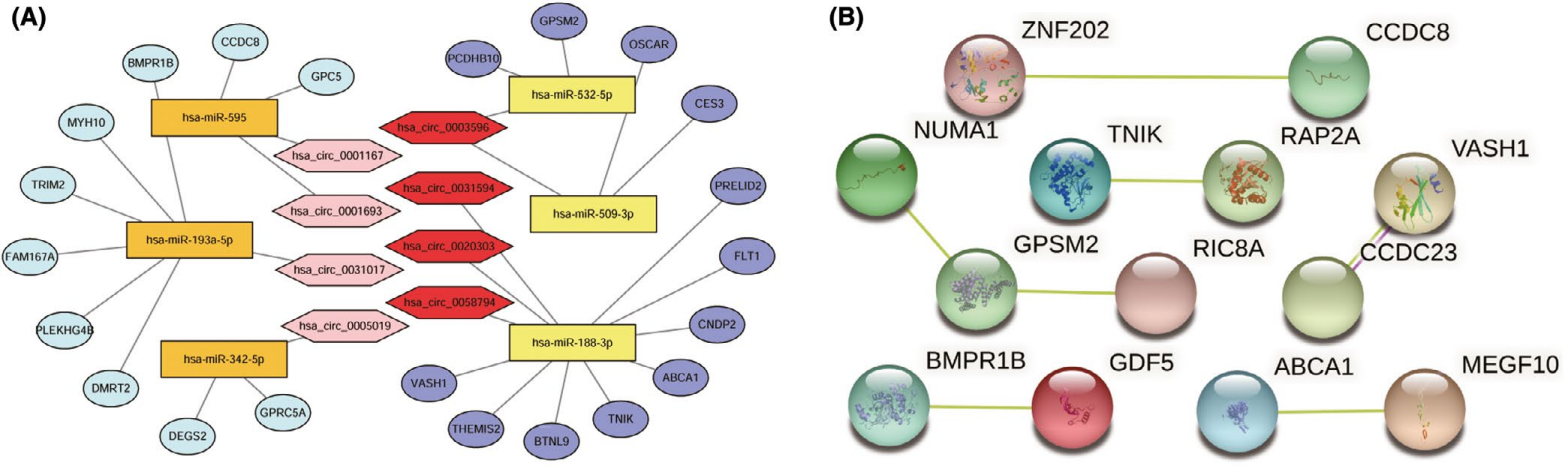
Construction of prognosis‐associated ceRNA subnet and PPI network. (A) The network consists of 8 circRNAs, 6 miRNAs, and 22 mRNAs. CircRNAs, miRNAs, and mRNAs are respectively represented by hexagons, rectangles, and ellipses. Relatively darker color indicates higher expression in the tumor tissues relative to normal tissues. (B) PPI network of genes in the prognosis‐associated subnet. The lines in different colors represent different relationships (Pink line: experimentally determined; Yellow line: textmining)

According to the 22 mRNAs, a prognosis‐associated ceRNA subnet was further constructed, which included 8 circRNAs, 6 miRNAs, and 22 mRNAs. The subnet is shown in Figure 5A.

### 3.5 Construction of PPI network

The 49 mRNAs of the ceRNA network and the 22 mRNAs of the prognosis‐associated subnet were applied to construct the PPI network via the Search Tool for the Retrieval of the Interacting Genes (STRING, https://string‐db.org/), which are shown in Figures 3B and 5B, respectively, and some disconnected nodes were hidden. PPI network showed that the proteins have more interactions among themselves than what would be expected for a random set of proteins of similar size, drawn from the genome. Such an enrichment indicated that the proteins are at least partially biologically connected, as a group.

### 3.6 Validation of hsa_circ_0001167/hsa‐miR‐595/CCDC8 prognosis‐associated axis in cell lines and tissues

By comparing the differential expression of the 22 genes in TCGA and the prognosis‐associated subnet, we finally selected seven genes with the same expression trend for subsequent studies (DEGS2, OSCAR, GPSM2, TRIM2, THEMIS2, MYH10, and CCDC8).

**FIGURE 6:**
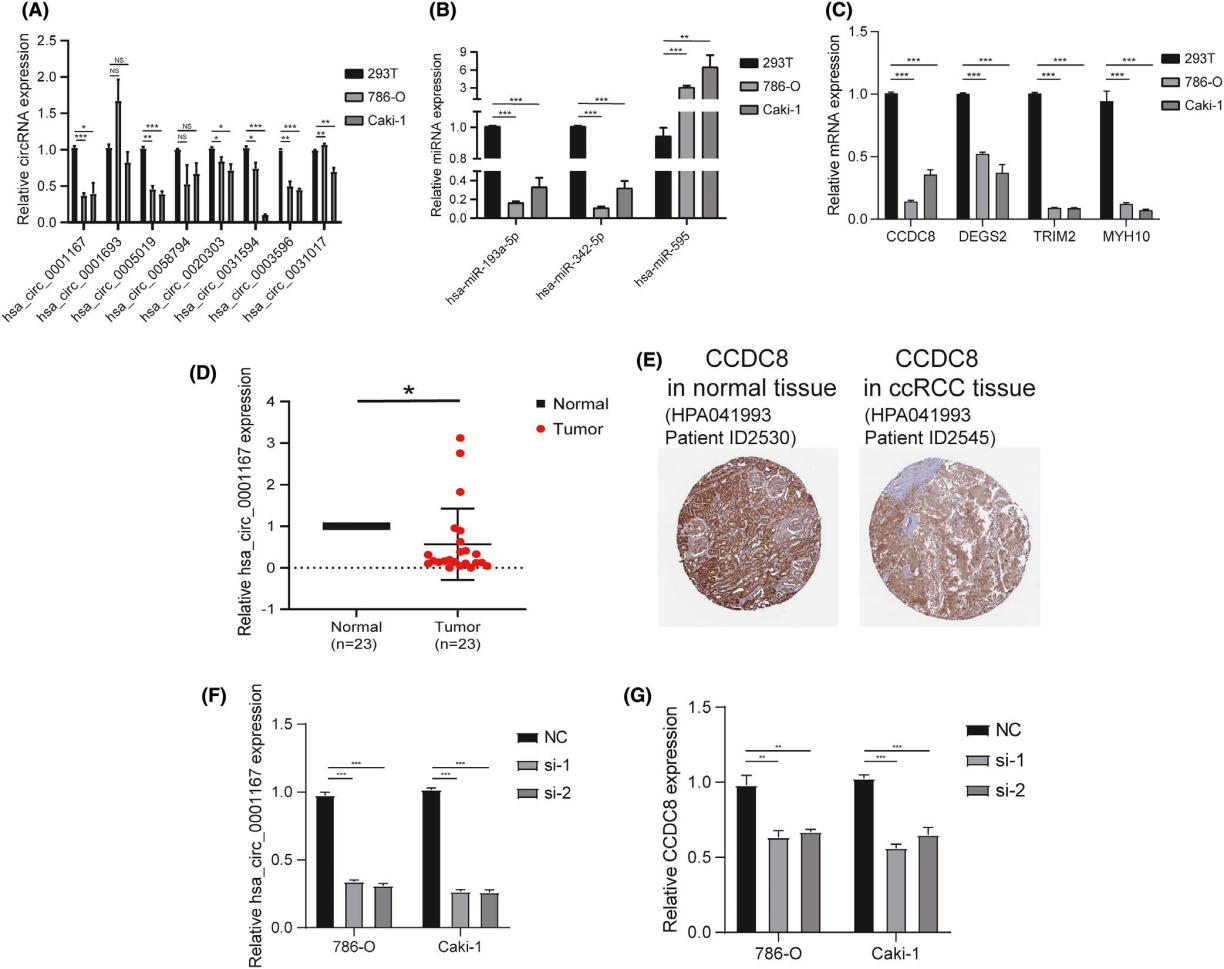
Validation of hsa_circ_0001167/hsa‐miR‐595/CCDC8 prognosis‐associated axis in cell lines and tissues. (A) Bar plot for the relative expression of eight circRNAs. (B) Bar plot for the relative expression of three miRNAs. (C) Bar plot for the relative expression of four mRNAs. (D) Dot plot for the relative expression of hsa_circ_0001167 in 23 pairs of tissues. (E) Immunohistochemical analysis of CCDC8 in ccRCC tissue and normal tissue from HPA database. (F) Expression of hsa_circ_0001167 in 786‐O and Caki‐1 after transfected with siRNA targeting the back‐splice region. (G) Expression of CCDC8 in 786‐O and Caki‐1 after transfected with siRNA targeting hsa_circ_0001167. NC: negative control; siRNA: small interfering RNA. NS means no significant, * means *p* value <0.05, ** means *p* value <0.01, and *** means *p* value <0.001

We identified the relevant circRNAs and miRNAs in our prognosis‐associated subnet based on the above‐mentioned seven genes and performed quantitative real‐time PCR (qRT‐PCR) to detect their expressions in the human kidney tumor cell lines 786‐O, Caki‐1 and the normal renal cell line HEK 293T. The relative expressions of six circRNAs (has_circ_0001167, has_circ_0005019, has_circ_0020303, has_circ_0031594, has_circ_0003596, and has_circ_0031017) in the tumor cell lines were significantly lower than that in HEK 293T cells, while another two circRNAs (has_circ_0001693 and has_circ_0058794) were no significant (Figure 6A). The results were compared with our prediction in the prognosis‐associated subnet and only three circRNAs (hsa_circ_0001167, hsa_circ_0005019, and hsa_circ_0031017) exhibited the same trend. We then measured the expression of related miRNAs (hsa‐miR‐595, hsa‐miR‐193a‐5p, hsa‐miR‐342‐5p) and mRNAs (CCDC8, TRIM2, DEGS2, MYH10) based on the above‐mentioned circRNAs in cell lines. As shown in Figure 6C, all the four mRNAs presented lower expression in Caki‐1 and 786‐O, which was consistent with our prediction. However, only hsa‐miR‐595 showed the same trend as our prognosis‐associated subnet (Figure 6B). In summary, the hsa_circ_0001167/hsa‐miR‐595/CCDC8 prognosis‐associated axis predicted by our bioinformatics technology was successfully validated at the expression level in cells.

We further collected 23 pairs of tumor and adjacent non‐tumorous tissues from ccRCC patients for qPCR. The expression of has_circ_0001167 was dramatically lower in ccRCC tissues than in adjacent‐tumor tissues (Figure 6D), which was the same as the results predicted by bioinformatics methods. Moreover, the Human Protein Atlas (HPA) database^23^, ^24^, ^25^ clearly revealed that the expression of coiled‐coil domain containing 8 (CCDC8) in ccRCC tissues was considerably lower (Figure 6E), which can also confirm our prediction.

To further verify our identification, we knocked down hsa_circ_0001167 in both 786‐O and Caki‐1 cells with small interfering RNA (siRNA) targeting the back‐splice region (Figure 6F). Down‐regulation of hsa_circ_0001167 significantly reduced the expression of CCDC8 in both 786‐O and Caki‐1 cells (Figure 6G). Taken together, the hsa_circ_0001167/hsa‐miR‐595/CCDC8 prognosis‐associated axis was confirmed.

## 4 DISCUSSION

Clear cell renal cell carcinoma is the most prevalent type of RCC, which is severely endangering human health.^26^, ^27^ Several studies have identified that the development of ccRCC involves a variety of gene expression disorders,^28^ with the circRNA/miRNA/mRNA axis playing an important role in regulating the expression of genes related to the prognosis of ccRCC.^29^, ^30^ For instance, Chen et al.^31^ revealed that the hsa_circ_001895/miR‐296‐5p axis promoted the expression of SOX12 so as to induce ccRCC progression. Zhu et al.^32^ demonstrated that circAKT1 enhanced the progression of ccRCC by targeting miR‐338–3p/CAV1 axis. Wang et al.^33^ reported that circDHX33 facilitated ccRCC progression by acting as a miR‐489‐3p sponge and up‐regulating MEK1 expression. Thus, the circRNA/miRNA/mRNA ceRNA network has the potential to predict the prognosis of ccRCC and help tailor more targeted treatments for patients.

Various bioinformatics methods were applied in previous studies. Wei et al.^34^ used a local ccRCC circRNA microarray and a metastatic ccRCC circRNA microarray to construct a ceRNA network. Bai et al.^35^ implied only one ccRCC circRNA microarray to conduct a ceRNA network. In these studies, only data from circRNA microarrays were analyzed. In the present study, we selected three independent GEO microarrays covering circRNAs, miRNAs, and mRNAs for multiple analyses. Besides, we implied adjusted *p* value (*q* value) to screen out differentially expressed circRNAs, miRNAs, and mRNAs to reduce false discovery rate, and the results were more accurate. We established a circRNA‐based ceRNA network covering 8 circRNAs, 6 miRNAs and 49 mRNAs. We further constructed a prognosis‐associated ceRNA subnet consisting of 8 circRNAs, 6 miRNAs, and 22 mRNAs. Among the eight circRNAs, only hsa_circ_0031594 has been mentioned in previous studies. Both Bai et al.^35^ and Franz et al.^36^ reported the differential expression of hsa_circ_0031594 between ccRCC tissues and matched normal tissues but neither was studied further. However, another seven circRNAs were annotated for the first time in the present study and needed to be further investigated.

MicroRNAs are a class of non‐coding RNAs with 18–25 nucleotides that have been proved to be associated with various BP of tumor progression through the regulation of oncogenes and antioncogenes.^37^ In the present study, we annotated six miRNAs in the prognosis‐associated ceNRA subnet and all have been reported in previous studies. Hsa‐miR‐532‐5p was found by Alizadeh et al.^38^ as a diagnostic marker for pancreatic cancer; Wu et al.^39^ reported that functional genetic mutations at the miRNA binding‐site such as hsa‐miR‐509‐3p in CD44 were relevant to the risk of colorectal cancer in Chinese populations; Banerjee et al.^40^ identified hsa‐miR‐188‐3p to be a biomarker of cervical cancer; hsa‐miR‐342‐5p was reported by Ascoli et al.^41^ to be a diagnostic and prognostic indicator of sarcoidosis; Lazzarini et al.^42^ found hsa‐miR‐595 to be differentially expressed between progenitors from normal myometrium and leiomyoma; Wu et al.^43^ reported that exosomal hsa‐miR‐193a‐5p could partially inhibit HBV replication and transcription. These lines of evidence suggest that the miRNAs we predicted by the bioinformatics methods are inextricably linked to various tumors.

With regard to the 22 genes included in our prognosis‐associated ceRNA subnet, all of them could be found in HPA, a public database of human proteins.^23^ Among the 22 genes, some were found to be definitively associated with tumor progression and prognosis. For example, Xiao et al.^44^ reported that the down‐regulation of TRIM2 affects ccRCC progression and predicts poor survival; Slattery et al.^45^ identified FLT1 as a biomarker of colorectal cancer about patients' survival; Takahashi et al.^46^ reported the prognostic significance of TNIK in colorectal cancer.

Previous studies identified that dysregulation of CCDC8 was associated with various cancers such as breast cancer, gastric cancer, and lung cancer.^47^, ^48^, ^49^ However, we are the first to report the association between CCDC8 and ccRCC. CCDC8 encodes a coiled‐coil domain‐containing protein. The encoded protein functions as a cofactor required for p53‐mediated apoptosis following DNA damage and may also play a role in growth through interaction with the cytoskeletal adaptor protein obscurin‐like 1. According to HPA, CCDC8 is a favorable prognostic marker in renal cancer, which is consistent with our prediction. With the help of STRING, a functional protein association database, we performed a PPI network to visualize the association between CCDC8 and ZNF202, a gene involved in the AMACR promoter CpG island deletion hotspot and a cis‐regulatory element controlling gene expression in the colon, as reported by Zhang et al.^50^

We are the first to perform a joint analysis of three independent GEO microarrays (circRNA microarray, miRNA microarray, and mRNA microarray) in a search to construct a ceRNA regulatory network and its prognosis‐associated subnet of ccRCC. However, there are also some inadequacies in our study. Firstly, our original data were obtained from public databases, some of which may not be updated in a timely manner. Secondly, the ceRNA network and its prognosis‐associated subnet need to be verified both in vitro and in vivo.

Multivariate analyses showed that CCDC8 was a vital prognostic factor, suggesting that the circRNA‐based hsa_circ_0001167/has‐miR‐595/CCDC8 axis might play an important role in the prognosis of ccRCC. This study may make contributions to decision‐making in the treatment of ccRCC patients.

## 5 CONCLUSION

Based on the analysis of three GEO microarrays and TCGA cases, we successfully constructed a ccRCC centered ceRNA network and its prognosis‐associated subnet. We further validated a hsa_circ_0001167/ has‐miR‐595/CCDC8 axis in renal cell lines, which may serve as prognostic indicators.

## CONFLICT OF INTEREST

All authors declare no conflicts of interest.

## ETHICS APPROVAL

All patients consented to an institutional review that allows comprehensive analysis of tissues samples (Ethics committee of Zhongda Hospital of Southeast University).

## Supporting information

Figure S1

Figure S2

Figure S3

Figure S4

Table S1

## Data Availability

The data sets used and analyzed during the study are available from the corresponding author on reasonable request.
