# Supplementary figures and images for: Construction of circRNA‐based ceRNA network and its prognosis‐associated subnet of clear cell renal cell carcinoma
Source: Cancer Med. 2021 Sep 27;10(22):8210–21. doi: 10.1002/cam4.4311 (PMC8607260; doi:10.1002/cam4.4311)

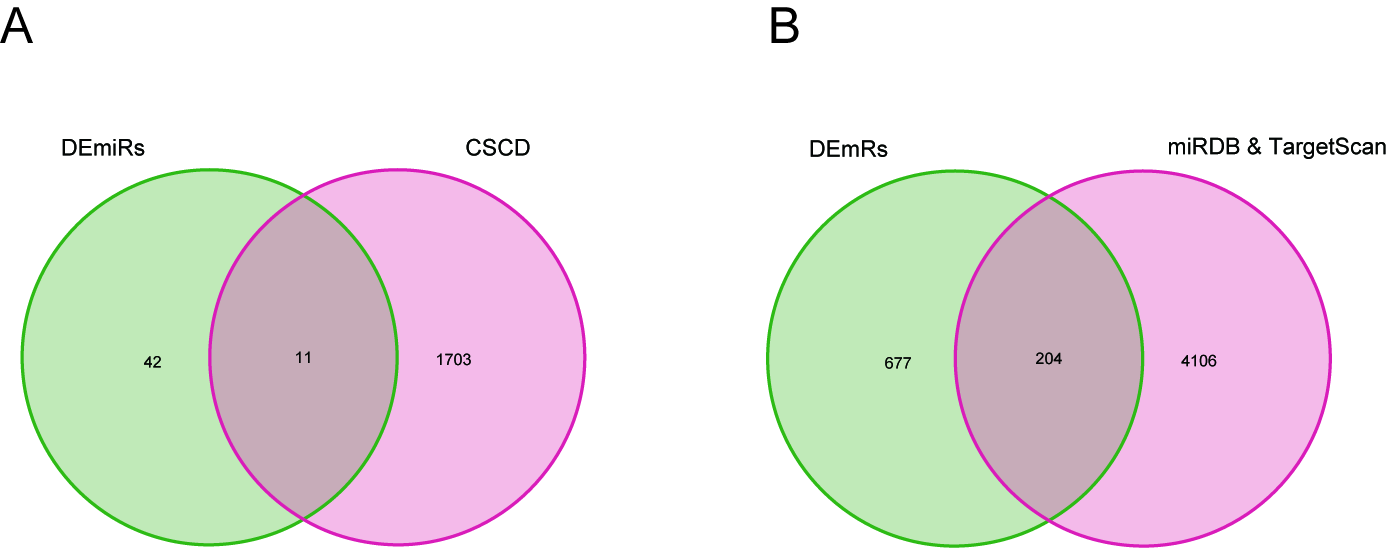

Supplement: Supplementary file 1 — Figure S1 [file CAM4-10-8210-s001.tif]

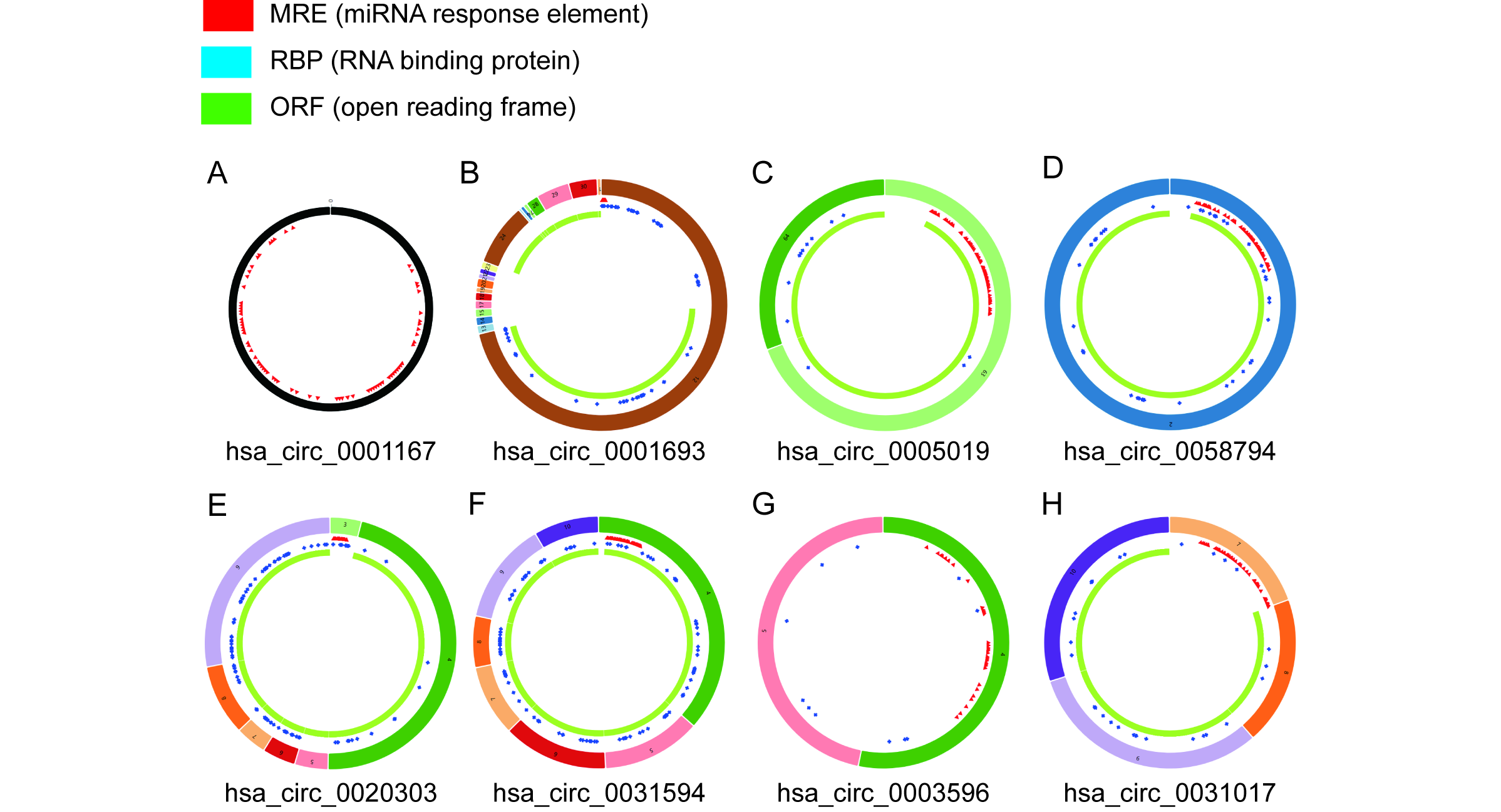

Supplement: Supplementary file 2 — Figure S2 [file CAM4-10-8210-s003.tif]

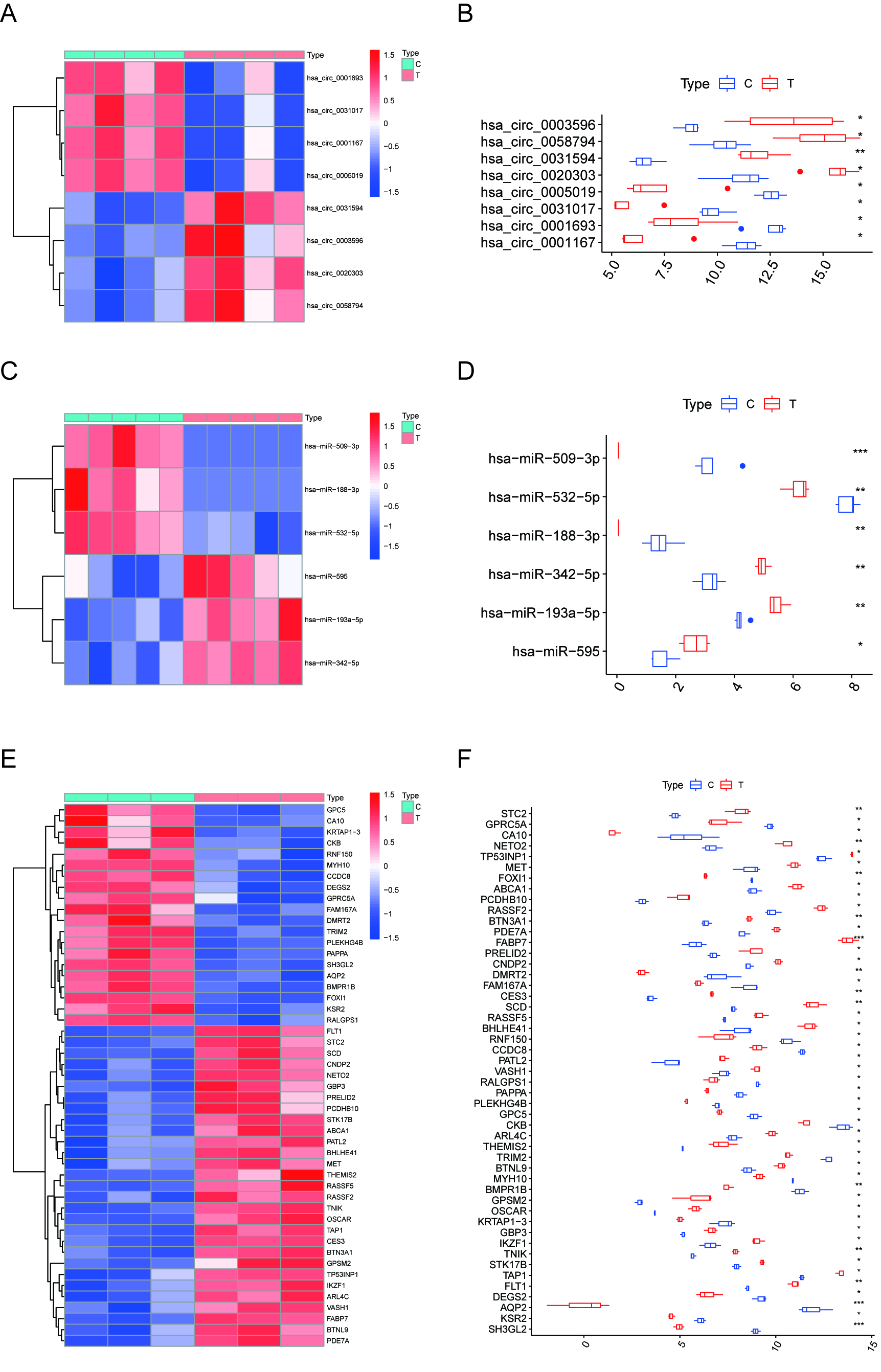

Supplement: Supplementary file 3 — Figure S3 [file CAM4-10-8210-s002.tif]

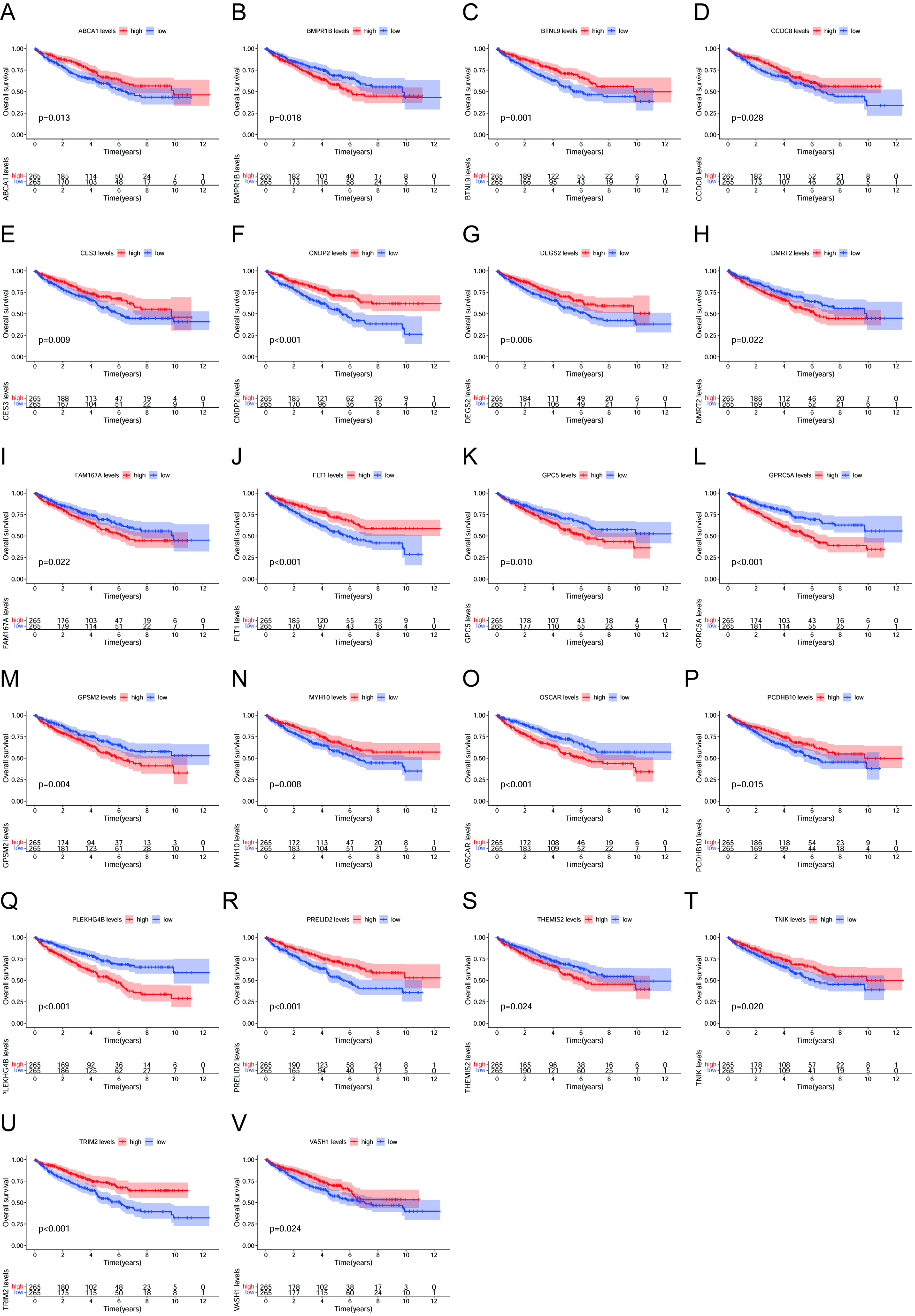

Supplement: Supplementary file 4 — Figure S4 [file CAM4-10-8210-s004.tif]
